# The Ethics of Chloroform Administration

**Published:** 1904-10-29

**Authors:** 


### Editor's Letter-Box.

[Our Correspondents are reminded that prolixity is a great bar to publication, and that brevity of style and conciseness of statement greatly facilitate early insertion.]

THE ETHICS OF CHLOROFORM ADMINISTRATION.

Sirs,—I would like to have the ethics of the administration of chloroform. Is a practitioner justified in giving it single-handed or by the hands of a nurse or a dispenser under his supervision while he operates, say in a bad forceps case or version? I hold that he is not, and have been contradicted by gentlemen who are in the habit of risking it. Your opinion would oblige.—Obstetric.

∴ It is always desirable that a second qualified medical man be called in to administer chloroform unless circumstances preclude such a course.—Ed. Hospital.

